# Management of well-appearing febrile infants aged 29–90 days: a multicenter survey on current practices and guideline adherence

**DOI:** 10.1186/s13052-026-02304-8

**Published:** 2026-07-31

**Authors:** Elisabetta Venturini, Gregorio Paolo Milani, Anna Comotti, Niccolò Parri, Agnese Tamborino, Elena Chiappini, Luisa Galli

**Affiliations:** 1 Paediatric Infectious Diseases Unit, Meyer Children’s Hospital IRCCS, Florence, Italy; 2 Pediatric Unit, Annunziata Hospital, Via Felice Migliori 1, 87100 Cosenza, Italy; 3 Department of Pharmacy, Health and Nutritional Sciences, University of Calabria, Rende, Italy; 4 Occupational Medicine Unit, Fondazione IRCCS Ca’ Granda Ospedale Maggiore Policlinico, Milan, Italy; 5 Department of Emergency Medicine and Trauma Center, Meyer Children’s Hospital IRCCS, Florence, Italy; 6 Department of Health Sciences, University of Florence, Florence, Italy

**Keywords:** Febrile infant, Fever without source, Pediatric emergency, Guideline adherence, Italy

## Abstract

**Background:**

Fever without a source in well appearing young infants remains a challenge in pediatric emergency departments (PEDs). Although evidence-based decision rules exist, limited data are available on how these patients are managed in daily clinical practice.

**Methods:**

We conducted a multicenter cross-sectional survey among pediatricians working in 20 Italian PEDs. The 22-item questionnaire explored the definition of “well appearing”, clinical assessment, diagnostic workup, use of standardized protocols, antibiotic prescribing, and self-reported competence in managing well-appearing febrile young infants aged 29–90 days. Descriptive statistics and Chi-square tests were used to compare responses by geographic area and physician seniority.

**Results:**

A total of 273 (response rate 84%) participated in the study. Half of them worked in Northern Italy, and 47% had < 5 years of experience. Standardized protocols were used by 56%, most commonly the Step-by-Step approach (28%) or local institutional guidelines (32%). Criteria used to define “well appearing” were highly heterogeneous. Urine dipstick testing was routinely performed by 80%, with culture obtained based on dipstick positivity in 66%. Inflammatory markers were routinely tested by 58%, and lumbar puncture was reserved for neurologic signs or clinical deterioration (63%). When inflammatory markers were within normal ranges, 67% discharged patients with close follow-up, while 83.5% reported using a conditional approach to antibiotic initiation guided by diagnostic findings (inflammatory markers and/or viral testing), mainly amoxicillin-clavulanate. Regional and seniority-related variability was significant.

**Conclusions:**

Italian PED physicians show moderate adherence to standardized protocols for managing well-appearing febrile young infants, but there is no shared definition of “well appearing”. These findings underscore the need for harmonized national guidelines and shared definitions to promote evidence-based care.

**Supplementary Information:**

The online version contains supplementary material available at https://doi.org/10.1186/s13052-026-02304-8.

## Introduction

Fever without a source (FWS) in young infants remains a challenging presentation in pediatric emergency departments (PEDs). FWS is defined as an acute febrile illness with onset of less than one week, in which no etiology is identified after a thorough history and physical examination and no localizing signs are present [1].

In well-appearing febrile infants aged 29–90 days, the risk of serious bacterial infections (SBI) is estimated at 5–15%, requiring a careful balance between under- and over-treatment [2–4]. Urinary tract infections account for most SBIs, whereas invasive bacterial infections (IBI) such as bacteremia and meningitis are substantially less common [5]. This epidemiologic distribution has important implications for clinical decision-making.

Several evidence-based clinical decision tools have been developed for risk stratification. Historical criteria such as Rochester and Philadelphia have largely been replaced by more recently validated approaches, including the Step-by-Step approach, the PECARN prediction rule, and the 2021 AAP guidelines [4]. However, variability persists in real-world implementation [6–8]. This variability includes diagnostic testing, adherence to standardized protocols, and antibiotic prescribing, and may be influenced by clinician experience, institutional guidelines, and regional practice [9–11]. Data describing current management of well-appearing febrile infants aged 29–90 days across European PEDs, including Italy, remain limited.

This study aimed to describe physicians’ self-reported clinical decision-making and management approaches for well-appearing febrile infants aged 29 to 90 days across Italian PEDs, using a multicenter cross-sectional survey.

## Methods

We conducted a multicenter cross-sectional survey among physicians working in Italian PEDs. Eligible participants were board-certified pediatricians with at least 6 months of pediatric emergency care experience. Pediatric residents and fellows were excluded to ensure that responses reflected clinicians with full clinical autonomy. The 20 participating centers are listed in Supplementary Table 1.

A structured 22-item questionnaire (italian language) was developed to assess management of well-appearing infants aged 29–90 days presenting with FWS. Because ‘well appearing’ is not uniformly operationalized across published pathways, the questionnaire did not mandate a specific scoring system; respondents were asked to apply their usual bedside assessment. This design choice may have increased between respondent variability in the interpretation of ‘well appearing’.

The survey was developed by four pediatricians (two pediatric infectious disease specialists and two pediatric emergency physicians), piloted in 18 pediatricians, and revised based on feedback. Test–retest assessment in four additional pediatricians showed intra-rater agreement > 98%.

The questionnaire addressed demographics, criteria used to assess appearance, use of standardized pathways, diagnostic testing (urine analysis, blood tests, respiratory viral testing), indications for lumbar puncture, antibiotic prescribing, and self-perceived competence. Given the survey-based design, responses reflect reported practice rather than directly observed clinical behavior.

The survey was distributed online in February 2025 using LimeSurvey. Participation was voluntary and anonymous. Descriptive statistics were used to summarize responses and are reported as frequencies and percentages. Responses were compared according to physician seniority (≤ 10 vs. > 10 years of experience) and geographic area (Northern, Central, and Southern Italy) using the Chi-square or Fisher’s exact test, as appropriate. For comparisons involving more than two groups, p-values were adjusted using the Bonferroni correction [12].

All analyses were performed using R software (version 4.5.1). A p-value < 0.05 was considered significant.

## Results

A total of 273 physicians (response rate 84%) participated. Most respondents were from Northern Italy (*N* = 138; 50%), followed by Central (*N* = 97; 36%) and Southern Italy (*N* = 38; 14%). Regarding experience, 47% (*N* = 128) had < 5 years of practice as board-certified pediatricians, 21% (*N* = 58) had 5–10 years, 20% (*N* = 55) had 11–20 years, and 12% (*N* = 32) had > 20 years.

### Definition of good general condition

The most commonly reported primary criterion for defining a well-appearing infant was use of a clinical scoring system (*N* = 115; 42%). Among those respondents, the Yale Observation Scale and the Pediatric Assessment Triangle were most frequently cited. Other commonly selected criteria included spontaneous movement and valid cry (*N* = 48; 18%), assessment of skin perfusion (*N* = 46; 17%), clinical intuition (*N* = 44; 16%), and absence of respiratory distress (*N* = 5; 2%). Routine use of standardized pathways (> 50% of cases or always) was reported by 56% (*n* = 153): 38% (*n* = 104) in > 50% of cases and 18% (*n* = 49) always. The remaining 44% (*n* = 120) reported pathway use in ≤ 50% of cases (24%, *n* = 65) or never (20%, *n* = 55). The Step-by-Step approach (*n* = 62; 28%) and local institutional guidelines (*n* = 87; 32%) were most commonly cited **(**Table 1**).**

**Table 1:** Criteria and protocols used in the clinical assessment of febrile infants

ITEMS	*n* (%)
**Main criterion to define good general condition (single-choice)**	
Absence of respiratory distress and normal oxygen saturation	5 (2)
Active spontaneous movements and vigorous crying	48 (18)
Pink skin color, well-perfused extremities, and absence of dehydration signs	46 (17)
Based on my clinical impression/intuition/experience	44 (16)
Based on parental reports	-
Use of a clinical scoring system (e.g.,Yale Observation Scale and the Pediatric Assessment Triangle)	115 (42)
Other	15 (5)
**Use of standardized protocols/flowcharts**	
Never	55 (20)
In <50% of cases	65 (24)
In >50% of cases	104 (38)
Always	49 (18)
**Type of standardized protocol used (if any, N=218) (multiple choice)**	
Rochester score	22 (10)
“Step-by-Step” approach score	62 (28)
Lab-score	3 (1)
Philadelphia score	27 (12)
American Academy of Pediatrics	9 (3)
American Heart Association (Pediatric advanced life support)	4 (1)
NICE guidelines	4 (1)
Other (e.g. internal protocols)	87 (32)

### Diagnostic testing

Most respondents (*N* = 220; 80%) reported routinely performing urine dipstick testing, most often using urine collection bags (*N* = 199; 73%). Urine cultures were typically obtained after a positive dipstick, with 66% (*N* = 172) preferring non-invasive collection methods (Table 2).

**Table 2:** Urine testing practices in the evaluation of febrile infants

ITEMS	n (%)
**Indications for urine dipstick test**	
Always, regardless of other investigations	220 (80)
Only in infants with negative chest X-ray (or other imaging) and, if performed, negative respiratory virus tests	18 (7)
Only in infants with negative respiratory virus tests, regardless of chest X-ray (or other imaging)	24 (9)
Other	11 (4)
**Method of urine collection for dipstick**	
Sterile urine bag	199 (73)
Nappy pad	13 (5)
Bladder catheterization	4 (1)
Stimulated clean-catch urine collection	11 (4)
Suprapubic aspiration	-
Sterile urine bag / nappy pad, and if positive, confirmation by invasive urine collection	46 (17)
**Performance of urine culture after dipstick**	
Always	17 (6)
If dipstick positive for leukocytes and nitrites	68 (25)
If dipstick positive for leukocytes only	174 (64)
Never	14 (5)
**Method of urine collection for culture (if yes, N=259)**	
Sterile urine bag / nappy pad	172 (66)
Bladder catheterization	47 (18)
Clean-catch midstream urine collection	40 (16)
Suprapubic aspiration	-

Routine respiratory viral testing was reported as never performed or performed in < 50% of cases by 48% (*N* = 103). Among physicians performing viral testing (*N* = 243), a limited panel (Influenza, RSV, SARS-CoV-2) was most common (54%; *N* = 132), followed by extended panels (37%; *N* = 90) (Table 3).

**Table 3:** Diagnostic approach to febrile infants

ITEMS	n (%)
**Routine respiratory virus testing**	
Never	30 (21)
In <50% of cases	73 (27)
In >50% of cases	59 (22)
Always	24 (9)
Only if recent close contacts with individuals with respiratory symptoms	53 (19)
Only during epidemic periods	34 (12)
**Type of respiratory viruses tested (if any, N=243) (multiple choice)**	
SARS-CoV-2	8 (3)
Influenza virus	4 (2)
Adenovirus	-
RSV (Respiratory Syncytial Virus)	9 (4)
Respiratory panel for Influenza, RSV, and SARS-CoV-2	132 (54)
Extended viral panel	90 (37)
**Blood tests for inflammatory markers (<24h fever)**	
Never	21 (8)
In <50% of cases	92 (34)
In >50% of cases	72 (26)
Always	88 (32)
**Which ones (if any, N=252) (multiple choice)**	
Complete blood count with differential	245 (97)
C-reactive protein (CRP)	248 (98)
Procalcitonin	193 (77)
Presepsin	2 (1)
**Blood culture request in ED (multiple choice)**	
Always	45 (16)
Depending on the results of inflammatory markers (if performed)	127 (47)
Based on the result of respiratory virus testing (if performed)	17 (6)
If urine dipstick positive for leukocytes/nitrites (if performed)	48 (18)
If urine dipstick negative for leukocytes/nitrites (if performed)	5 (2)
If chest X-ray (or other imaging) does NOT show pneumonia (if performed)	8 (3)
If chest X-ray (or other imaging) shows pneumonia (if performed)	25 (9)
If empirical antibiotic therapy is planned, regardless of other findings	23 (8)
Never	17 (6)
**Indications for lumbar puncture**	
Always	1 (0.4)
Only in infants developing signs of meningeal irritation, central nervous system involvement, or deterioration of general conditions during observation	171 (63)
If blood tests suggest a high risk of severe/invasive bacterial infection, even in the absence of meningeal signs or central nervous system involvement	101 (37)

Inflammatory markers were requested in > 50% of cases by 58% (*N* = 160). CRP (98%) and CBC with differential (97%) were most frequently used. Blood cultures were ordered based on inflammatory markers results by 47% (*N* = 127) and routinely by 16% (*N* = 45). Lumbar puncture was reserved for neurological signs or clinical deterioration by 63% (*N* = 171); the remaining 37% reported basing this decision primarily on laboratory results even without clinical suspicion.

### Clinical management and antibiotic prescribing

After normal test results and in the presence of reliable caregivers, 67% (*N* = 181) reported discharge with home monitoring and follow-up within 12–24 h. Additionally, 83.5% (*N* = 165) reported a conditional approach in which antibiotic initiation at discharge is guided by diagnostic findings (including inflammatory markers and/or rapid respiratory viral testing); when antibiotics were prescribed, the most commonly reported agent was amoxicillin–clavulanate **(**Table 4).

**Table 4:** Clinical management, empirical treatment, and self-perceived competence in febrile infants

ITEMS	n (%)
**Management after normal tests and compliant caregivers**	
Discharge with home monitoring by family	16 (6)
Discharge with home monitoring and follow-up visit within 12–24 hours	181 (67)
Hospital admission for observation in the emergency department, with possible repetition of tests after 12–24 hours	57 (21)
Hospital admission to the pediatric ward	12 (6)
Other	7 (2)
**Frequency of re-assessments during observation (if observation, N=57)**	
Every hour	7 (12)
Every 2–3 hours	29 (51)
Every 4–6 hours	11 (19)
Every 6–12 hours	3 (5)
Every 12–24 hours	-
Reassessments without defined intervals	7 (12)
**Empirical antibiotics for discharged patients (if discharged, N=197)**	
Always	3 (1.5)
Conditional—initiated only if investigations suggest bacterial infection (e.g., inflammatory markers and/or rapid viral testing)	165 (83.5)
Never	29 (15)
**Type of empirical oral antibiotic used (if any, N=168)**	
Amoxicillin	72 (43)
Amoxicillin-clavulanic acid	96 (57)
Cephalosporin	-
Macrolide	-
**Empirical antibiotics for patients under observation (if any, N=57) (multiple choice)**	
Always	4 (7)
In all patients in whom inflammatory markers were not performed	-
Depending on the results of inflammatory markers if performed	50 (88)
Depending on the results of respiratory virus testing if performed	2 (3)
Never	1 (2)
**Type of empirical antibiotic used during observation (if any, N=56)**	
Ampicillin or Amoxicillin	13 (23)
Ampicillin-sulbactam	8 (14)
Amoxicillin-clavulanic acid	9 (16)
Ampicillin + Gentamicin	9 (16)
Third-generation cephalosporin	17 (30)
Piperacillin-tazobactam	-
**Self-rated competence in managing infants with fever without source**	
Very adequate	21 (8)
Adequate	241 (88)
Inadequate	11 (4)
Very inadequate	-

An alternative approach was ED observation (*N* = 57; 21%), with reassessment every 2–3 h in 51%. During observation, 88% reported prescribing empiric antimicrobials based on inflammatory marker results, with variable agent selection: one-third third-generation cephalosporins, one-third aminopenicillin with a beta-lactamase inhibitor, and 23% aminopenicillin alone.

Most physicians rated their competence as adequate (88%) or very adequate (8%). Only 4% (*N* = 11) felt inadequately prepared.

### Differences by geographic area and physician seniority

Significant regional differences were observed. In Northern Italy, 27% used sterile urine bags with invasive confirmation, compared with 7% in Central Italy and none in Southern Italy (*p* < 0.001). Conversely, in Southern Italy, 32% never performed urine cultures after a positive dipstick test, versus 2% and 0% in Central and Northern Italy, respectively (*p* < 0.001). Bladder catheterization was significantly more frequent in Northern Italy (32%) than in Central and Southern Italy (4% each; *p* < 0.001). Respiratory viral testing also varied significantly (*p* < 0.001), with limited panels more commonly used in Northern Italy (61%). Differences were also observed according to physician seniority. Regarding inflammatory markers in infants with 24 h fever, 11% of junior physicians (< 10 years) reported never performing them, versus 0% of senior physicians (*p* = 0.04). Junior physicians reported significantly lower self-perceived competence (5% “very adequate” vs. 14% seniors, *p* = 0.003). All physicians reporting inadequate competence had ≤10 years’ experience.

## Discussion

This multicenter survey highlights significant variability in clinical decision-making for well-appearing febrile infants aged 29–90 days across Italian PEDs, particularly regarding standardized protocols, diagnostic testing, and antibiotic prescribing.

Only 56% of physicians routinely reported using standardized protocols. Despite the availability of international tools (Rochester, Philadelphia, Step-by-Step, PECARN and 2021 AAP guideline), internal institutional guidelines were most commonly cited, suggesting reliance on local protocols rather than international standards [4]. This pattern is consistent with European evidence: a Spanish Delphi study identified only four quality indicators achieving ≥ 95% consensus for managing febrile infants ≤ 90 days [13]. The need for formal consensus processes underscores the absence of universally accepted standards across European PEDs.

The most common criterion for defining good general condition was use of clinical scoring systems (42%). However, the concept of “well-appearing infant” inherently introduces subjectivity, which may contribute to assessment variability. Reported “scoring systems” included tools developed for rapid recognition of critical illness (Pediatric Assessment Triangle) and older illness-appearance scores (Yale Observation Scale), underscoring the lack of a shared operational construct of “well appearing” in this age group.

Overall, the observed heterogeneity in the operationalization of “well appearance” limits between-center comparability and may contribute to variability in downstream diagnostic and therapeutic thresholds. This issue is internationally recognized: the AAP guideline explicitly notes that “well appearing” is often treated as a binary construct but remains subjective and lacks an adequate definition, and recommends not applying the algorithm when clinicians are uncertain about an infant’s well appearance [4]. Similarly, the ACEP clinical policy highlights heterogeneity in definitions and the lack of consensus regarding the most useful clinical prediction approach [14]. These findings support the need for an international consensus process to develop a shared operational definition and/or a standardized bedside assessment framework for “well-appearing” febrile infants.

The observed regional variability reflects a broader European phenomenon. The MOFICHE study documented substantial practice variation across 12 European EDs, with diagnostic test utilization ranging from 14% to 83%, antibiotic treatment from 23% to 54%, and full guideline adherence in only 15% of cases [4]. The discrepancy between self-reported practices and actual clinical behavior is an important consideration; MOFICHE findings suggest that self-reported adherence may overestimate actual guideline-concordant care [4].

Most respondents (80%) routinely performed urine dipstick testing, consistent with evidence reporting 94% sensitivity and 91% specificity for UTI diagnosis in febrile infants ≤ 60 days [15] However, most physicians reported sending urine cultures from bag-collected samples without catheterization confirmation. Only 27% in Northern Italy confirmed positive bag specimens with invasive methods, compared with 7% in Central Italy and none in Southern Italy. According to AAP guidance, bag-collected cultures have false-positive rates of 88–99% [16]. This regional disparity may partially explain geographic differences in antibiotic prescribing.

Routine respiratory virus testing reported as never performed or performed in < 50% of cases by 48% of respondents. Current guidelines recommend that viral testing should not alter the initial diagnostic pathway for infants > 28 days, but may help individualize management decisions during peak viral seasons [4, 17–19]. Infants with documented viral infections have significantly lower SBI rates [20–22]. However, viral positivity does not exclude concurrent bacterial infections and none of the major validated pathways include viral testing as a criterion [6, 8]. A 2014 systematic review found insufficient evidence supporting routine rapid viral testing for reducing antibiotic use [17]. Our data suggest that respiratory panels may represent a potential target for standardization in future guidelines [4, 10]. 

Regarding lumbar puncture, 37% of physicians reported basing this decision primarily on laboratory results even in the absence of overt clinical suspicion. However, the survey quantified the frequency of diagnostic test use but did not capture the specific clinical triggers (e.g., history, physical examination findings, or risk-stratification thresholds) guiding test ordering; accordingly, our data describe practice patterns but do not allow inference on the appropriateness of testing or diagnostic stewardship. Current ACEP guidance highlights that no predictors adequately identify which well-appearing full-term febrile infants aged 29–90 days require cerebrospinal fluid testing; therefore, lumbar puncture may still be considered, while deferral is a reasonable option in infants diagnosed with a viral illness provided close follow-up is ensured and antibiotics are withheld unless another bacterial source is identified [14]. In contrast, the PECARN clinical prediction rule for febrile infants ≤ 60 days identified a low-risk group using urinalysis, absolute neutrophil count, and procalcitonin, with no missed cases of bacterial meningitis in derivation and validation cohorts, supporting shared decision-making and a more selective approach to lumbar puncture in low-risk infants [23].

Our survey did not assess whether decisions were stratified by infant age (29–60 vs. 61–90 days) or fever degree. Recent evidence suggests no temperature cut-off can safely rule out IBI: in a Spanish multicenter study, 46.7% of IBIs were diagnosed in patients with temperature ≤ 38.6 °C [12]. Future surveys should explore whether Italian physicians apply age- and temperature-stratified approaches.

A conditional approach to antibiotic prescribing was commonly reported: 83.5% (*N* = 165) indicated that antibiotic initiation at discharge was guided by diagnostic findings (inflammatory markers and/or rapid respiratory viral testing), rather than prescribed routinely. When antibiotics were prescribed, the most commonly reported agent was amoxicillin–clavulanate (57%) and amoxicillin (43%) (Table 4). This finding is consistent with the between-centre variability and frequent broad-spectrum use reported across European EDs in PERFORM/MOFICHE, where overall antibiotic prescribing was 31.9% and broad-spectrum prescribing among antibiotic recipients was 52.1% [24]. Although our survey did not capture the clinical indications for each prescription, contemporary pathways support withholding antibiotics in selected low-risk, well-appearing infants when urinalysis and inflammatory markers are reassuring, provided close follow-up is ensured.

The frequent adoption of conditional strategies based on inflammatory markers (83.5%) suggests that actual antibiotic prescribing depends on diagnostic findings. However, the survey did not capture the actual antibiotic prescribing rate, the specific thresholds used to interpret inflammatory markers, or patient-level outcomes. Several factors may contribute to variability in antibiotic use, including inconsistent urine collection techniques leading to false positives, limited protocol adherence, precautionary approaches, heterogeneous interpretation of inflammatory markers or empirical coverage pending viral results [4, 25].

While procalcitonin > 0.5 ng/mL has demonstrated high sensitivity (83%) and negative predictive value (99.8%) for ruling out invasive bacterial infections in well-appearing febrile infants ≤ 60 days, its positive predictive value remains low (7.4%), potentially leading to unnecessary investigations and antibiotic treatments [25]. Combined use of clinical assessment and inflammatory markers within validated risk-stratification pathways may improve decision-making and reduce inappropriate prescribing [4, 25].

Less-experienced physicians (< 10 years of practice) were more likely to report never obtaining inflammatory markers in infants with 24 h fever (11% vs. 0% among more experienced physicians), and all physicians reporting inadequate competence had < 10 years of experience. This experience-related gap underscores the need for targeted educational interventions and mentorship.

This study has several limitations. First, because this was a self-reported survey, the findings are prone to both social desirability bias and recall bias, which may substantially affect the validity of our results. Social desirability bias may have led physicians to report management strategies that align more closely with guideline-concordant “ideal” practices rather than reflecting actual clinical behavior, thereby systematically overestimating real-world adherence to recommended pathways and underestimating the true extent of practice variation. This bias is particularly concerning in the context of antibiotic prescribing and diagnostic testing decisions, where physicians may be aware of “correct” guideline recommendations but face different pressures and constraints in actual clinical practice. Additionally, recall bias may have influenced responses, as the survey inquired about “usual” or “routine” practices rather than case-specific decision-making processes. This approach may have elicited oversimplified or stereotyped responses that do not fully capture the complexity and variability of actual bedside management, where decisions are often influenced by multiple contextual factors including patient presentation severity, parental anxiety, time of day, availability of follow-up, and individual risk tolerance. The combination of these biases means that our findings likely represent an idealized version of practice patterns rather than actual clinical behavior, and the true magnitude of inter-center variation and non-adherence to guidelines may be substantially greater than reported. Furthermore, the survey quantified the frequency of diagnostic test use but did not capture the specific clinical triggers guiding test ordering; therefore, our data describe reported practice patterns but do not allow inference on the appropriateness of testing or diagnostic stewardship. This limitation is particularly relevant in light of MOFICHE [4], which documented substantial between-centre variation in the evaluation and management of febrile children in EDs and suggested incomplete adherence to recommended pathways in routine practice. Accordingly, our results should be interpreted as reported practices and attitudes rather than an objective measure of bedside management, and the true magnitude of practice variation and non-adherence may be greater than reported.

Second, participation was limited to 20 centers and may not fully represent all Italian pediatric emergency settings. The survey assessed reported decision-making strategies (e.g., conditional approach to antibiotic prescribing) but did not capture actual antibiotic prescribing rates, the specific clinical or laboratory thresholds used to initiate antibiotics, or patient-level outcomes. The absence of patient-level data represents a critical limitation that prevents correlation of physician self-reported behaviors with actual patient outcomes and precludes any causal inference regarding the impact of reported practices on clinical endpoints such as missed serious bacterial infections, unnecessary hospitalizations, or antibiotic-related adverse events. Without objective data on what physicians actually do (as opposed to what they report doing) and how their decisions affect patient outcomes, we cannot determine whether reported practice variations translate into differences in patient safety, quality of care, or resource utilization. This gap between self-reported practice and observed behavior is well-documented in the medical literature and may be particularly pronounced in high-stakes clinical scenarios such as febrile infant management, where cognitive biases, medicolegal concerns, and system-level factors may drive decision-making in ways that physicians themselves may not fully recognize or report. Additionally, the survey did not stratify analyses by age subgroups (29–60 vs. 61–90 days), despite evidence that IBI prevalence differs significantly between these groups [14]. Because the survey did not include age-stratified items, we were unable to assess whether diagnostic and management strategies differed by age subgroup, which may mask important age-related variations in practice patterns. Future studies should prospectively collect data on actual prescribing rates, clinical indications, and outcomes to quantify the gap between reported strategies and observed practice, ideally using chart review or electronic health record data linked to patient outcomes. Third, results may not be generalizable to infants with systemic illness signs. The survey was not specifically designed to evaluate antibiotic prescribing in detail; heterogeneity may reflect differences in clinical interpretation and awareness of the declining incidence of Listeria [26]. The study did not assess within-center variability, which may be as large or larger than between-center variation, and did not collect patient-level outcomes. The lack of standardized quality indicators for febrile infant management in Italian EDs limits benchmarking against evidence-based standards and makes it difficult to assess whether observed practice variations represent appropriate individualization of care or problematic deviations from best practice [13]. 

## Conclusion

This study reveals considerable heterogeneity in the management of well-appearing febrile infants aged 29–90 days across Italian PEDs. While many physicians report using standardized protocols, important gaps remain in the definition of “well appearing,” consistency of diagnostic testing, and evidence-based decision-making. These findings highlight the need for concrete steps toward practice harmonization.

To address these gaps, Italian pediatric societies should collaborate to develop and disseminate national consensus guidelines that incorporate validated risk stratification tools such as Step-by-Step or PECARN, along with standardized definitions of clinical appearance. Such guidelines would provide a common framework while allowing for local adaptation based on available resources. Implementation should be supported through practical toolkits that include clinical pathways, decision aids, and educational materials to facilitate adoption in diverse PED settings.

Particular attention should be given to standardizing the approach to viral testing, as our findings demonstrate substantial variation in which tests are performed, when they are ordered, and how results influence clinical decisions regarding antibiotic use and hospitalization. Clear, evidence-based recommendations in this area could reduce unnecessary testing while ensuring appropriate risk stratification.

To monitor progress and identify opportunities for improvement, establishment of a national registry or quality improvement network would enable tracking of key metrics including rates of lumbar puncture, hospitalization, antibiotic use, and missed serious bacterial infections. Such data would support continuous quality improvement and help identify best practices that could be disseminated across centers.

Future studies should triangulate survey findings with prospective audits and chart reviews to quantify discrepancies between reported and observed practice, and to evaluate whether harmonized pathways are associated with improved clinical outcomes, reduced practice variation, and more appropriate resource utilization.

## Supplementary Information

Below is the link to the electronic supplementary material.

Supplementary Material 1

Supplementary Material 2

## Data Availability

The datasets generated during and/or analysed during the current study are not publicly available but are available from the corresponding author on reasonable request.

## Declarations

### FEBRIOUT study group: FEBRIle infant withOUT a source

Elena Altieri^1^, Lorenza Baroni^2^, Roberta Caiazzo^3^, Crescenzo Coppola^3^, Giulia Dal Canto^4^, Margherita Del Bene^5^, Giada Di Pietro^6^, Federica Forlanini^7^, Chiara Gabrielli^8^, Luisa Giannone^9^, Ilaria Liguoro^10^, Giulia Lorenzetti^11^, Caterina Marabotto^12^, Carmela Mercogliano^13^, Sara Monaco^14^, Cristina Moracas^5^, Marco Antonio Motisi^15^, Paola Musso^16^, Francesco Nunziata^13^, Victoria Rinaldi^8^, Alessia Sallemi^7^, Marco Sarno^17^, Agnese Tamborino^18^, Marco Zaramella^19^

^1^ASST Grande Ospedale Metropolitano Niguarda, Milan, Italy

^2^Neonatology and Neonatal Intensive Care Unit, ASMN Reggio Emilia, Reggio Emilia, Italy

^3^Pediatric Infectious Disease Unit, Luigi Sacco Hospital, University of Milan, Milan, Italy

^4^Pediatric Unit, Imola Hospital, Imola, Italy

^5^Pediatric Infectious Disease Unit, Department of Maternal and Child health, University Hospital “Federico II”, Naples, Italy

^6^Department of Pathophysiology and Transplantation, Pediatric Highly Intensive Care Unit, Fondazione IRCCS Ca’ Granda Ospedale Maggiore Policlinico, University of Milan, Milan, Italy

^7^Paediatric Department, Buzzi Children’s Hospital, Milan, Italy

^8^Division of Pediatrics, Nuovo Ospedale San Giovanni Battista, Foligno; Ospedale Civile San Matteo degli Infermi, Spoleto, Italy

^9^ASST Spedali Civili, Brescia, Italy

^10^Division of Pediatrics, University Hospital of Udine, Udine, Italy

^11^Infectious Diseases Unit, Bambino Gesù Children’s Hospital, IRCCS, Rome, Italy

^12^Department of Pediatrics, Bambino Gesù Children’s Hospital, IRCCS, Palidoro, Rome, Italy

^13^General Paediatrics and Immuno-Rheumatology Unit, National Specialty Hospital Santobono Pausilipon, Naples, Campania, Italy

^14^Pediatric Unit, Fidenza Hospital, Local Health Authority, Parma, Italy

^15^Pediatrics and Neonatology unit, Maternal-Infant Department, San Giuseppe Hospital, Empoli, Italy

^16^Department of Pediatrics, SS. Annunziata Hospital, Savigliano (CN), Italy

^17^Chronic Diseases, Hepatology and Nutrition Unit, Santobono-Pausilipon Children’s Hospital, Naples, Italy

^18^Paediatric Infectious Disease Unit, Meyer Children’s Hospital IRCCS, Florence, Italy

^19^Pediatrics and Neonatology unit, Ospedale Unico della Versilia, Lido di Camaiore (LU), Italy

## Abbreviations

AAP: American Academy of Pediatrics
ACEP: American College of Emergency Physicians
CBC: Complete Blood Count
CI: Confidence Interval
CRP: C–Reactive Protein
ED: Emergency Department
FWS: Fever Without a Source
IBI: Invasive Bacterial Infection
MOFICHE: Management and Outcome of Fever in Children in Europe
PECARN: Pediatric Emergency Care Applied Research Network
PED: Pediatric Emergency Department
RSV: Respiratory Syncytial Virus
SARS: CoV–2–Severe Acute Respiratory Syndrome Coronavirus 2
SBI: Serious Bacterial Infection
UTI: Urinary Tract Infection
WHO: World Health Organization
