# Supplementary material for: Management of well-appearing febrile infants aged 29–90 days: a multicenter survey on current practices and guideline adherence
Source: Ital J Pediatr. 2026 Jul 31;52:157. doi: 10.1186/s13052-026-02304-8 (PMC13725946; doi:10.1186/s13052-026-02304-8)
Supplement: Supplementary file 1 [file 13052_2026_2304_MOESM1_ESM.docx]

**Supplementary online material**

**Supplementary table 1**. List of participating centers

| **CENTERS** | **N** |
| --- | --- |
| Ospedale di Empoli, Empoli | 10 |
| Ospedale Bambino Gesù di Palidoro, Rome | 30 |
| Ospedale Ca’ Granda, De Marchi, Milan | 14 |
| Ospedale di Castellammare di Stabia | 6 |
| Ospedale Sacco, Milan | 10 |
| Ospedale di Reggio Emilia | 7 |
| Ospedale Giovanni Battista, Foligno | 11 |
| Ospedale Versilia di Camaiore, Lucca | 8 |
| Ospedale Santobono, Naples | 23 |
| Ospedale Metropilitano di Niguarda, Milan | 24 |
| Ospedale Vittore Buzzi, Milan | 21 |
| Spedali Civili, Brescia | 15 |
| Ospedale Bambin Gesù Gianicolo, Rome | 4 |
| Azienda Sanitaria Universitaria Friuli Centrale – ASUFC, Udine | 15 |
| Ospedale Meyer, Firenze | 25 |
| Ospedale di Salerno | 7 |
| Ospedale SS. Annunziata, Savigliano (Cuneo) | 11 |
| Ospedale di Pisa | 5 |
| Ospedale di Fidenza | 11 |
| Ospedale di Perugia | 4 |
| Ospedale di Imola | 10 |
| Ospedale Civico di Cristina-Palermo | 2 |

**Supplementary Table 2. Sample characteristics**

| **Geographic area**  Lombardy  Piemonte  Emilia-Romagna  Friuli Venezia Giulia  Tuscany  Umbria  Lazio  Campania  Sicily  Northern Italy  Centre Italy Southern I | 84 (31)  11 (4)  28 (10)  15 (5)  48 (18)  15 (5)  34 (12)  36 (13)  2 (1)  138 (50)  97 (36)  38 (14) |
| --- | --- |
| **Experience as a pediatric specialist**  <5 years  5–10 years  11–20 years  20 years | 128 (47)  58 (21)  55 (20)  32 (12) |
