# Supplementary material for: Management of well-appearing febrile infants aged 29–90 days: a multicenter survey on current practices and guideline adherence
Source: Ital J Pediatr. 2026 Jul 31;52:157. doi: 10.1186/s13052-026-02304-8 (PMC13725946; doi:10.1186/s13052-026-02304-8)
Supplement: Supplementary file 2 [file 13052_2026_2304_MOESM2_ESM.doc]

Prof. Gregorio Paolo Milani

Pediatric Unit, Foundation Istituto di Ricovero e Cura a Carattere Scientifico (IRCCS) Ca' Granda Ospedale Maggiore Policlinico, Milan, Italy

Dear Editor,

We wish to submit the original article entitled “**Management of Well-Appearing Febrile Infants Aged 29–90 Days: A Multicenter Survey on Current Practices and Guideline Adherence** for consideration by *Italian Journal of Pediatrics.* We confirm that this work is original and has not been published elsewhere, nor is it currently under consideration in another journal. All authors have seen and approved the manuscript. Each author has contributed significantly to the work. A study group has been added, as there were a large group of participants.

We believe that this manuscript fits the scope of the journal.

We have no conflicts of interest to disclose.

Thank you for your consideration of this manuscript.

Sincerely,

Prof. Gregorio Paolo Milani

milani.gregoriop@gmail.com
